# Evaluation of the clinical value of serum inflammatory mediators in assessing sepsis severity

**DOI:** 10.12669/pjms.42.1.12765

**Published:** 2026-01

**Authors:** Xinghua Li, Shiwu Zhang

**Affiliations:** 1 Xinghua Li Tianjin Medical University, Tianjin 300070, Tianjin, China; 2 Shiwu Zhang Department of Pathology, Tianjin Union Medical Center, Tianjin 300121, Tianjin, China

**Keywords:** Sepsis, Inflammatory mediator, Disease severity, Prognostic prediction

## Abstract

**Objective:**

To evaluate the clinical utility of serum inflammatory mediators in assessing the severity of sepsis and predicting patient prognosis.

**Methodology:**

A retrospective analysis was conducted on 72 patients diagnosed with sepsis and admitted to the Tianjin Union Medical Center between January 2024 to January 2025, who were stratified into three groups based on disease severity: sepsis(*n*= 24), severe sepsis(*n*= 24) and septic shock(*n*= 24). An additional 24 individuals without sepsis were recruited as the control group. Within 24 hours of hospital admission, serum levels of tumor necrosis factor-alpha (TNF-α), interleukin-10 (IL-10) and heparin-binding protein(HBP) were measured, analyzed for their correlation with Acute Physiology and Chronic Health Evaluation II (APACHE-II) scores and their predictive performance for 28-day mortality.

**Results:**

Significant differences were observed in serum TNF-α, IL-10, HBP levels and APACHE-II scores across the four groups(all *P*< 0.05). All three sepsis subgroups showed significantly elevated levels of TNF-α, IL-10, HBP and higher APACHE-II scores compared with the control group (all *P* < 0.05). Moreover, a graded increase in these biomarkers and APACHE-II scores was observed across the sepsis subgroups, following the trend: septic shock > severe sepsis > sepsis, with all pairwise comparisons reaching statistical significance(P< 0.05, respectively). When stratified into survival and non-survival groups, the non-survivors exhibited significantly higher levels of TNF-α, IL-10, HBP and APACHE-II scores(*P*< 0.005, respectively).

**Conclusion:**

Serum levels of TNF-α, IL-10 and HBP are positively correlated with sepsis severity. The combined measurement of these inflammatory mediators can provide a robust and objective approach for evaluating disease severity and predicting prognosis in patients with sepsis.

## INTRODUCTION

Sepsis is a life-threatening condition characterized by organ dysfunction resulting from a dysregulated host response to infections. It affects approximately 30 million people worldwide each year and is responsible for nearly six million deaths, making it a major global health burden and a central challenge in critical care medicine.^1^ The pathophysiology of sepsis is primarily driven by immune-inflammatory dysregulation, where excessive activation of immune cells leads to the overproduction of inflammatory mediators. Septic shock is an acute circulatory disorder based on sepsis and shock caused by hypoperfusion. This cascade results in microcirculatory disturbances, endothelial injury and ultimately, multiple organ failure. Inflammatory mediators are not only central drivers of sepsis progression but also serve as critical biomarkers for assessing disease severity.^2^

Traditionally, the severity of sepsis has been evaluated using clinical scoring systems and conventional biomarkers. However, these methods are often limited by low sensitivity and specificity, as well as delayed responsiveness, which may hinder timely therapeutic intervention.^3^ In recent years, growing interest in serum inflammatory mediators has opened new avenues for the stratified management of sepsis. These mediators include pro-inflammatory cytokines such as interleukin-10 (IL-10), tumor necrosis factor-alpha (TNF-α) and heparin-binding protein (HBP), which amplify the inflammatory response; immunoregulatory molecules such as programmed death-1/programmed death ligand 1 (PD-1/PD-L1), which signal immune exhaustion and increased risk of secondary infection;^4^ and endothelial injury markers like HBP, which disrupt the vascular barrier.

The dynamic changes in these inflammatory mediators reflect not only the real-time intensity of the immune response but also the phase of immune dysregulation, thereby offering valuable guidance for personalized immunomodulatory therapy.^5^ Moreover, multiplex detection of these mediators, especially when integrated with emerging technologies, further enhances their diagnostic and prognostic utility. Taken together, serum inflammatory mediators serve as a “molecular mirror” of sepsis pathophysiology and hold irreplaceable value in disease stratification, prognosis prediction and therapeutic decision-making.^6^ On this basis, the present study aimed to evaluate the clinical significance of serum inflammatory mediators in assessing the severity of sepsis.

## METHODOLOGY

This retrospective study included 72 patients diagnosed with sepsis and admitted to the Tianjin Union Medical Center between January 2024 and January 2025. Based on the severity of the illness, patients were divided into three groups: sepsis group, severe sepsis group and septic shock group, with 24 patients in each. Additionally, patients were further categorized into a survival group and a non-survival group according to their 28-day mortality outcome. A control group consisting of 24 non-septic patients hospitalized during the same period was also included for comparison. Clinical data were collected, including patient demographics (age, sex), underlying conditions, body temperature, APACHE-II scores and laboratory test results.

### Ethical Approval:

The study was approved by the Institutional Ethics Committee of Baoding NO.1 Central Hospital (No.:[2024]126; Date: November 27, 2024) and written informed consent was obtained from all participants.

### Inclusion criteria:

- Diagnosis of sepsis according to the Third Edition of International Consensus Definitions for Sepsis and Septic Shock.
- Age ≥ 18 years with complete clinical data.
- Onset of symptoms within 24 hours before admission.
- Normal mental status at enrollment.

### Exclusion criteria:

- Hospital stay ≤ 24 hours.
- Concurrent malignancies or autoimmune diseases.
- Recent use of immunosuppressants or biologics.
- Pregnancy or lactation.

Within 24 hours of hospital admission, five mL of venous blood was drawn from each patient with sepsis and placed into sterile tubes. After resting at room temperature for 30 minutes, samples were centrifuged at 4,000 rpm for 10 minutes. One milliliter of serum was harvested and transferred into Eppendorf tubes, sealed and stored at -80°C until analysis. For control subjects, five mL of fasting morning venous blood was collected and processed using the same protocol.

*Biomarker Assays:* After thawing at room temperature, serum concentrations of TNF-α, IL-10 and HBP were measured using enzyme-linked immunosorbent assay (ELISA) based on the double-antibody sandwich technique. All procedures were performed in accordance with the manufacturer’s instructions and conducted by certified laboratory personnel.

### Outcome Measures:

- Serum levels of TNF-α, IL-10 and HBP were compared among all patient groups.
- APACHE-II scores were compared across the sepsis, severe sepsis and septic shock groups. The APACHE-II scoring system consists of three components, namely acute physiological score, age score and chronic health evaluation, with a maximum score of 71. Lower scores are indicative of a better prognosis.
- Serum TNF-α, IL-10 and HBP levels, as well as APACHE-II scores, were compared between the survival and non-survival groups.

### Statistical Analysis:

All statistical analyses were performed using SPSS 26.0. The Shapiro-Wilk test was used to assess the normality of continuous variables. Data with a normal distribution are expressed as mean ± standard deviation (*χ̅*±*S*), while non-normally distributed data are presented as median (1^st^ quartile, 3^rd^ quartile) (*M* [*Q*_1_-*Q*_3_]). Independent sample t-tests were used for comparisons between two groups and one-way analysis of variance was used for comparisons among multiple groups. Pairwise comparisons between groups were conducted using the Student-Newman-Keuls method. Pearson correlation analysis was employed to assess the relationship between serum levels of TNF-α, IL-10, HBP and APACHE-II scores. Receiver operating characteristic (ROC) curve analysis was conducted to evaluate the predictive value of TNF-α, IL-10 and HBP for 28-day mortality in patients with sepsis, with survivors defined as negative cases and non-survivors as positive cases. A *P*-value < 0.05 was considered statistically significant.

## RESULTS

In the sepsis group, there were 11 males and 13 females, aged 30 to 80 years, with a mean age of 50.75 ± 12.43 years. In the severe sepsis group, 12 males and 12 females were included, aged 33 to 77 years, with a mean age of 52.58 ± 8.38 years. The septic shock group comprised 10 males and 14 females, aged 30 to 79 years, with a mean age of 50.96 ± 9.35 years. The control group included 12 males and 12 females, aged 32 to 81 years, with a mean age of 53.04 ± 10.08 years.

Significant differences were observed among the four groups in serum TNF-α, IL-10 and HBP levels, as well as APACHE-II scores (*P <* 0.05, respectively). Compared with the control group, the sepsis, severe sepsis and septic shock groups had significantly elevated levels of TNF-α, IL-10 and HBP, as well as higher APACHE-II scores (*P <* 0.05, respectively). Moreover, all four markers showed a stepwise increase with disease severity, with the increase being most pronounced in the septic shock group, followed by the severe sepsis group and the sepsis group; between-group comparisons demonstrate statistically significant differences (*P <* 0.05, respectively) (Table-I).

**Table-I:** Intergroup comparison of serum TNF-α, IL-10, HBP levels and APACHE-II scores (*χ̅*±*S*).

Group	n	TNF-α (fmol/ml)	IL-10 (ng/ml)	HBP (ng/ml)	APACHE-II score
Sepsis	24	6.35±0.47	41.63±1.29	59.52±1.33	25.75±3.44
Severe sepsis	24	9.55±0.76	53.23±1.62	63.84±1.10	32.96±4.75
Septic shock	24	12.17±1.18	69.13±2.17	79.07±1.16	40.17±4.85
Control group	24	4.55±0.54	35.01±1.54	22.14±1.02	8.67±2.62
*F-value*		445.261	1890.058	10411.513	269.918
*P-value*		<0.001	<0.001	<0.001	<0.001

Among the 72 patients with sepsis, 12 died within 28 days, yielding a case fatality rate of 16.67%. Based on 28-day outcomes, patients were classified into a survival group (*n =* 60) and a non-survival group (*n =* 12). The non-survival group exhibited significantly higher serum levels of TNF-α, IL-10 and HBP, as well as higher APACHE-II scores compared with the survival group (*P <* 0.005, respectively) (Table-II).

**Table-II:** Comparison of serum TNF-α, IL-10, HBP levels and APACHE-II scores between survivors and non-survivors (*χ̅*±*S*).

Group	n	TNF-α (fmol/ml)	IL-10 (ng/ml)	HBP (ng/ml)	APACHE-II score
Survival	60	8.85±2.34	52.34±10.67	65.66±7.82	31.88±7.26
Non-survival	12	11.76±2.20	65.94±7.86	76.52±5.77	39.42±4.87
*t-value*		3.970	4.184	4.561	3.434
*P-value*		<0.001	<0.001	<0.001	<0.001

**Fig.1:**
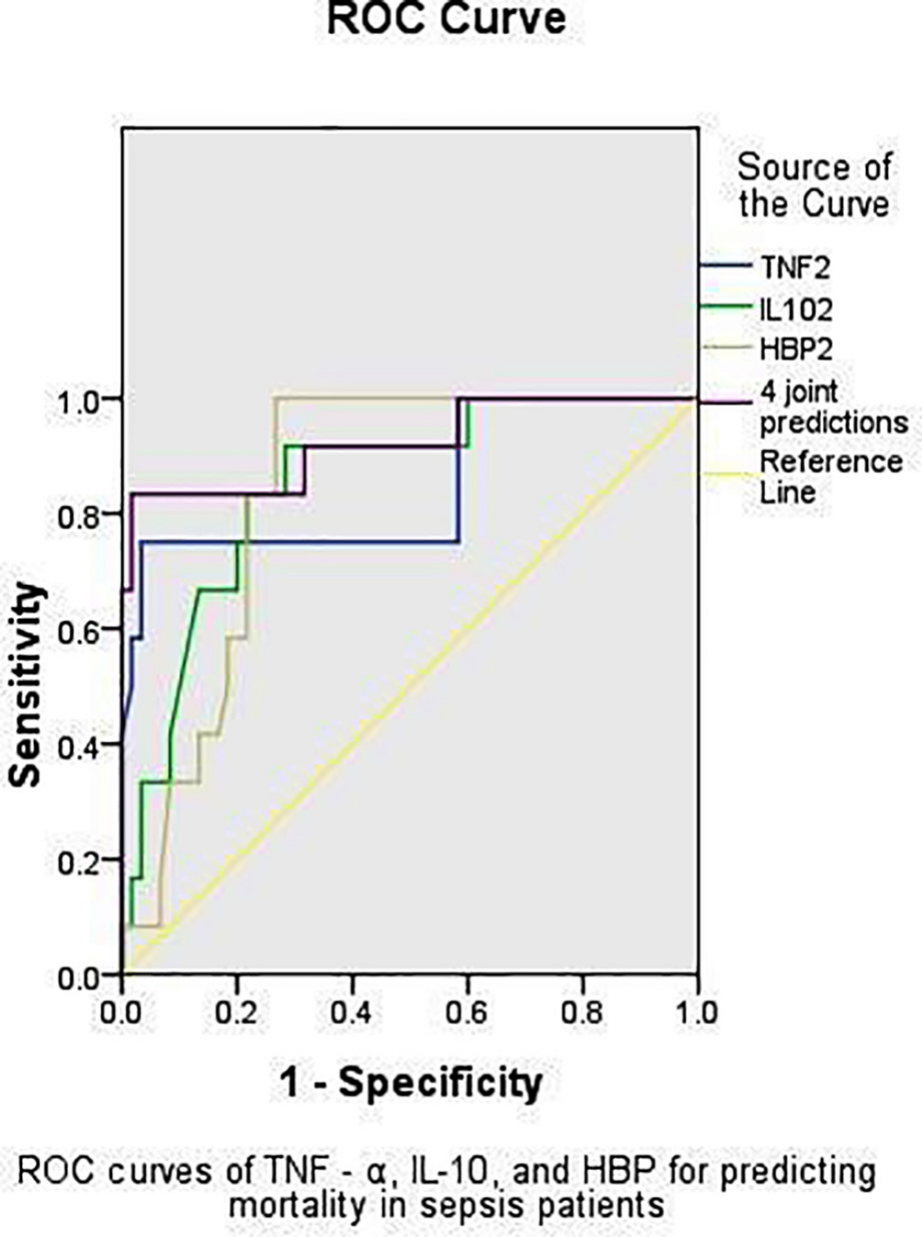
ROC curves of TNF-α, IL-10 and HBP for predicting 28-day mortality in patients with sepsis.

Pearson correlation analysis revealed that serum levels of TNF-α, IL-10 and HBP were positively correlated with APACHE-II scores in patients with sepsis. (Table-III). ROC curve analysis demonstrated that the multiplex detection of TNF-α, IL-10 and HBP yielded an area under the curve (AUC) of 0.922 for predicting mortality in patients with sepsis, significantly higher than those of any individual biomarker (*P <* 0.05, respectively) (Table-IV) and (Fig.1).

**Table-III:** Correlation between serum TNF-α, IL-10, HBP levels and APACHE-II scores.

	TNF-α	IL-10	HBP
r-value	0.686	0.801	0.759
P-value	<0.001	<0.001	<0.001

**Table-IV:** Predictive performance of TNF-α, IL-10 and HBP for 28-day mortality in patients with sepsis.

Marker	AUC	95% CI	P-value	Youden Index	Optimal Cut-off	Sensitivity (%)	Specificity (%)
TNF-α	0.847	0.701-0.992	<0.001	0.717	12.355 (fmol/ml)	75.0	96.7
IL-10	0.851	0.743-0.958	<0.001	0.617	62.090 (ng/ml)	83.3	76.7
HBP	0.842	0.753-0.931	<0.001	0.733	64.255 (ng/ml)	100.0	73.3
Multiplex detection	0.922	0.000-1.000	<0.001	0.817		83.3	98.3

## DISCUSSION

This study systematically evaluated the clinical value of serum inflammatory mediators, including TNF-α, IL-10 and HBP, in the stratification and prognostication of patients with sepsis. The results demonstrated a stepwise increase in the serum levels of TNF-α, IL-10 and HBP, as well as in APACHE-II scores, with the progression from sepsis to severe sepsis and ultimately to septic shock, with statistically significant differences between groups (all *P <* 0.001). When stratified by 28-day survival outcomes, all measured markers were significantly lower in the survivor group compared with the non-survivor group (*P <* 0.001, respectively). These findings are highly consistent with results from previous clinical studies,^7^-^9^ suggesting that the severity of organ dysfunction directly influences the magnitude of inflammatory mediator release.^10^ Notably, this study is the first to validate the coordinated elevation pattern of TNF-α, IL-10 and HBP in patients with sepsis within a Chinese population. Moreover, it quantitatively established the characteristic serum concentrations of these three mediators at the septic shock stage, providing an important clinical reference for diagnostic and therapeutic protocols in Chinese and broader East Asian populations. Pearson correlation analysis further confirmed strong positive correlations between the serum TNF-α, IL-10 and HBP levels and APACHE-II scores, underscoring the utility of these biomarkers in assessing disease severity and predicting outcomes in patients with sepsis. These findings align with the emerging clinical paradigm that proposes the “inflammatory mediator profile as a real-time molecular mirror of the pathophysiological progression of sepsis”. 11,12

Despite the high mortality rate associated with sepsis, there is still a lack of accurate prognostic factors with both high sensitivity and specificity for predicting patient outcomes.^13^ In this study, ROC curve analysis was used to evaluate the predictive value of serum TNF-α, IL-10 and HBP levels for 28-day mortality in patients with sepsis. The results demonstrated that the multiplex detection of TNF-α, IL-10 and HBP yielded significantly higher prognostic accuracy than any single marker alone. These findings are consistent with previous clinical studies suggesting that multiplex detection can improve the precision of sepsis outcome prediction.^14^

Importantly, this study is the first to identify optimized cutoff values for the combined use of TNF-α (>12.355 fmol/ml), IL-10 (>62.090 ng/ml) and HBP (>64.255 ng/ml) in a Chinese patient population, achieving a sensitivity of 83.3% and specificity of 98.3% for predicting sepsis-related mortality. Notably, HBP alone exhibited a sensitivity of 100%, which is significantly higher than the 85% sensitivity reported in earlier clinical studies.^15^,^16^ This underscores the unique prognostic value of HBP as an early sentinel biomarker; when HBP exceeds 64.255 ng/ml, it can almost entirely identify patients at high risk of death, highlighting the critical need for prompt initiation of intensive treatment in these individuals. However, its specificity (73.3%) is lower than that of the multiplex detection approach. Therefore, concurrent measurement of TNF-α, IL-10 and HBP is essential to further improve the accuracy of mortality prediction in patients with sepsis.

This study is the first to propose and establish a high-risk cutoff value of HBP *>*64.255 ng/ml in a Chinese sepsis population, addressing the current gap in diagnostic standards for this biomarker in Chinese patients. Notably, this threshold is higher than that reported in European studies (>50 ng/ml), suggesting that racial or ethnic differences may influence the reference ranges of biomarkers.^17^ Furthermore, our findings revealed a 30% surge in IL-10 levels in the septic shock group compared with the severe sepsis group, a magnitude significantly higher than previously reported in existing studies.^18^-^20^ The underlying mechanisms for this marked elevation remain to be elucidated. The proposed multiplex detection model may compensate for the time lag associated with the APACHE-II scoring system, enabling stratification at admission. Its high specificity helps avoid overtreatment and reduces healthcare burden, while its operational simplicity makes it particularly suitable for use in resource-limited primary care settings.

### Limitations:

First, it is a single-center retrospective analysis with a relatively small sample size, which may introduce selection bias. Second, comorbidities such as pulmonary infections, diabetes and hypertension were not matched between groups, potentially affecting baseline comparability. Third, only three inflammatory mediators (*i.e*., TNF-α, IL-10, HBP) were included, limiting the breadth of biomarker coverage. Additionally, only a single serum sample collected within 24 hours of admission was analyzed, which precluded dynamic assessment of biomarker trajectories and their correlation with treatment response. The interactions among these mediators were also not explored.

### Recommendations:

Future studies should involve prospective cohort designs with larger sample sizes and a broader panel of inflammatory markers to validate and expand upon these findings.

## CONCLUSIONS

This study demonstrates that the serum levels of TNF-α, IL-10 and HBP are effective biomarkers for assessing disease severity and prognosis in patients with sepsis. Their combined use can significantly enhance the accuracy of mortality risk prediction.

### Authors’ Contributions:

**XL:** Study design, literature search, manuscript writing, data collection, data analysis, interpretation and critical review, is responsible and accountable for the accuracy or integrity of the work. **SZ:** Manuscript revision and validation, critical analysis. All authors have read and approved the final manuscript.
